# Effective cataract surgical coverage: An indicator for measuring quality-of-care in the context of Universal Health Coverage

**DOI:** 10.1371/journal.pone.0172342

**Published:** 2017-03-01

**Authors:** Jacqueline Ramke, Clare E. Gilbert, Arier C. Lee, Peter Ackland, Hans Limburg, Allen Foster

**Affiliations:** 1 School of Social Sciences, Faculty of Arts and Social Sciences, University of New South Wales, Sydney, New South Wales, Australia; 2 School of Population Health, University of Auckland, Auckland, New Zealand; 3 Department Clinical Research, Faculty Infectious & Tropical Diseases, London School of Hygiene and Tropical Medicine, London, United Kingdom; 4 International Agency for the Prevention of Blindness, London, United Kingdom; 5 Health Information Services, Nijenburg 32, Grootebroek, Netherlands; LV Prasad Eye Institute, INDIA

## Abstract

**Objective:**

To define and demonstrate *effective cataract surgical coverage (eCSC)*, a candidate UHC indicator that combines a coverage measure (cataract surgical coverage, CSC) with quality (post-operative visual outcome).

**Methods:**

All Rapid Assessment of Avoidable Blindness (RAAB) surveys with datasets on the online RAAB Repository on April 1 2016 were downloaded. The most recent study from each country was included. By country, cataract surgical outcome (CSO_Good_, 6/18 or better; CSO_Poor_, worse than 6/60), CSC (operated cataract as a proportion of operable plus operated cataract) and eCSC (operated cataract and a good outcome as a proportion of operable plus operated cataract) were calculated. The association between CSC and CSO was assessed by linear regression. Gender inequality in CSC and eCSC was calculated.

**Findings:**

Datasets from 20 countries were included (2005–2013; 67,337 participants; 5,474 cataract surgeries). Median CSC was 53.7% (inter-quartile range[IQR] 46.1–66.6%), CSO_Good_ was 58.9% (IQR 53.7–67.6%) and CSO_Poor_ was 17.7% (IQR 11.3–21.1%). Coverage and quality of cataract surgery were moderately associated—every 1% CSC increase was associated with a 0.46% CSO_Good_ increase and 0.28% CSO_Poor_ decrease. Median eCSC was 36.7% (IQR 30.2–50.6%), approximately one-third lower than the median CSC. Women tended to fare worse than men, and gender inequality was slightly higher for eCSC (4.6% IQR 0.5–7.1%) than for CSC (median 2.3% IQR -1.5–11.6%).

**Conclusion:**

eCSC allows monitoring of quality in conjunction with coverage of cataract surgery. In the surveys analysed, on average 36.7% of people who could benefit from cataract surgery had undergone surgery and obtained a good visual outcome.

## Introduction

Quality-of-care encompasses many clinical and non-clinical dimensions [1] and is one of the objectives embodied by the concept of Universal Health Coverage (UHC), together with equity in access and financial protection.[2] *Effectiveness* is considered one of seven attributes that define quality of health care, and is defined as the degree to which attainable health improvements are realized.[3]

Historically, coverage indicators focused on access coverage, reflecting the proportion of a population needing a service who used it. As early as 2001 the World Health Organization (WHO) recognized the importance of monitoring quality in addition to access, and promoted the routine assessment of *effective coverage* to reflect the proportion of a population needing a service who used it *and* obtained the desired result.[4, 5] Initially effective coverage was not widely adopted, possibly due to perceived complexity and the absence of data for its calculation.[6] Effective coverage has received more interest in the context of UHC,[7, 8] and the ability to measure effective coverage was included as a criterion for UHC tracer indicators in the 2016 World Health Statistics.[5]

For many health interventions assessing quality in addition to access coverage is not straight forward. For most of the current UHC tracer indicators effective coverage cannot be measured [5]; for other interventions the calculation of effective coverage relies on the use of intervention inputs as proxy indicators for the quality of the intervention outcomes.[9, 10] Cataract surgery is an intervention for which effective coverage can be measured using the intervention outcome—visual acuity assessment has been cited as a biomarker to indicate quality in the context of UHC,[7, 8, 11] and assessment of post-operative visual acuity can provide an indication of the effectiveness of cataract surgery at restoring vision.

Cataract is a clouding of the lens of the eye which reduces visual acuity. It is the leading cause of blindness globally, affecting 10.8 million people in 2010, while a further 35.2 million people had moderate or severe visual impairment due to cataract.[12] Age-related cataract occurs as a result of denaturation of lens proteins and is currently thought to be irreversible. With aging of the global population[13] the number of people with vision-impairing cataract will increase unless cataract services improve in terms of access, output and quality. Accordingly, control of blindness and visual impairment due to cataract is a priority in the World Health Organization’s (WHO) current “Universal Eye Health: a global action plan 2014–2019” which was endorsed at the 66^th^ World Health Assembly”[14, 15] (hereafter called the UEH Action Plan).

The only treatment currently available for cataract is surgical removal of the opaque lens with implantation of an artificial intra ocular lens (IOL) to correct the refractive error (termed aphakia). The use of IOLs is now universally accepted as the treatment of choice, giving immediate and better visual rehabilitation than aphakic correction with spectacles. Cataract surgery is a cost-effective intervention[16] that usually restores sight.[17] It can also improve quality-of-life,[18–20] time-use,[20] and social status[19] and positively impacts on poverty alleviation.[18–20] These strengths and benefits have contributed to the inclusion of cataract surgery in recent essential surgery lists,[21, 22] as well as in the proposed initial surgical package for UHC.[23] Cataract surgery can, however, be associated with poor visual outcomes for several reasons. Firstly, another eye condition was known to be present before surgery, such as corneal scarring, where surgery can improve vision but not to ‘normal’. Alternatively, cataract surgery may reveal retinal or optic nerve disease which was not suspected before surgery. Second, the surgery could be complicated, and third there may be longer-term complications such as corneal oedema, retinal detachment, and thickening of the lens capsule, all of which are more frequent following suboptimal surgery. Lastly, there may be inadequate optical correction despite use of an IOL. In a study of almost 5,200 cataract operations in eight centres in Africa and Asia, almost three-quarters of poor outcomes were attributed to short and long term surgical complications or inadequate optical correction.[24] Visual acuity after cataract surgery does, therefore, to a large extent reflect the quality of cataract surgical services and post-operative care.

The UEH Action Plan includes an access coverage indicator for cataract services in the form of *cataract surgical coverage (CSC)*, which measures the number of people in a defined population with operated cataract as a proportion of those having operable plus operated cataract.[25] The WHO identified CSC as a promising UHC indicator in its inaugural UHC monitoring report, recognizing its value beyond monitoring coverage of eye services, as a means of measuring access to services for the elderly more generally.[26] Despite the strengths of CSC, it does not provide any indication of the quality of cataract services and so by itself is insufficient to track progress towards UEH.

Fortunately, it is possible from data already collected in population-based visual impairment surveys to calculate an indicator that combines CSC with a measure of cataract surgery quality, in the form of visual outcome in the operated eye (‘cataract surgical outcome’). We have called this indicator *Effective Cataract Surgical Coverage* (eCSC), and the aim of this paper is to define and demonstrate eCSC as a candidate UHC tracer indicator.

## Methods

### Data source and study selection

The data used in this analysis were sourced from the online Repository of Rapid Assessment of Avoidable Blindness (RAAB) surveys (hereafter called the Repository; http://www.raabdata.info/). RAAB is a cross-sectional population-based survey of blindness and visual impairment in people aged 50 years and above that has been validated (for blindness) against population-based surveys.[27, 28] RAAB surveys are restricted to those aged 50 years and above as the prevalence of blindness is highest in this age group,[27] so the sample size is smaller, and the survey shorter and less expensive than surveys including all ages.[29] RAAB uses standard examination methods, and the software, which is available freely for download,[30] includes a data entry module with a comprehensive validation system and a standardized and automated analysis package.

RAAB is listed as a preferred methodology in the UEH Action Plan[14] and is now the most commonly implemented blindness survey methodology. RAAB was piloted in 2005, and by April 1 2016, 266 separate RAAB surveys from 74 countries had been registered on the Repository. Once surveys are registered, principal investigators are invited to allow their completed dataset to be freely available for download from the Repository. To construct the sample for this analysis, all RAAB datasets available on the Repository on April 1 2016 were downloaded. For countries with more than one dataset available, the most recent was selected for inclusion. Where numerous surveys had been conducted in the same country in the same year, one was randomly selected for inclusion. Ethics approval was not sought for this analysis as the datasets were anonymized and publicly available.

### Definitions and variables

*Blindness* was defined as visual acuity of worse than 3/60 in the better eye and *severe visual impairment* (SVI) was defined as visual acuity of worse than 6/60 but 3/60 or better in the better eye.

*Operated cataract* was defined as the presence of pseudophakia (implanted IOL) or aphakia (no IOL) on internal eye examination.

Visual acuity assessment commonly includes measurement of presenting visual acuity (i.e. with spectacles if normally worn) as well as pinhole acuity. The use of a pinhole corrects vision loss that is due to refractive error, which can be corrected by spectacles. In RAAB surveys pinhole acuity is used to identify operable cataract when pinhole acuity remains poor in the presence of an opaque lens. In many low and middle income countries, as in high income countries, cataract surgery is now often offered before someone becomes blind. Therefore, in this analysis, *operable cataract* was defined as pinhole visual acuity of SVI or blindness (less than 6/60) where the principal cause was cataract. The analysis can be repeated using other visual acuity cut-offs for operable cataract as appropriate (e.g. <3/60, < 6/18).

*Cataract surgical coverage* (CSC) was defined as the number of people in a defined population with operated cataract as a proportion of those having operable plus operated cataract[25] (i.e. pinhole visual acuity worse than 6/60; CSC_persons <6/60_).

CSC=[(x+y)/(x+y+z)]*100(%)

where

*x* = individuals with unilateral pseudo/aphakia (i.e. operated cataract) and operable cataract in the other eye;
*y* = individuals with bilateral pseudo/aphakia, regardless of visual acuity;
*z* = individuals with bilateral operable cataract.

*Cataract surgical outcome* (CSO) was defined as presenting visual acuity in the operated eye of a person who had undergone unilateral cataract surgery, and presenting visual acuity in the better eye of a person who had undergone bilateral cataract surgery. The WHO guidelines[31] were used to categorise CSO into CSO_Good_ (6/18 or better), CSO_Borderline_ (worse than 6/18 to 6/60) or CSO_Poor_ (worse than 6/60).

*Effective cataract surgical coverage* (eCSC) measures the number of people in a defined population with operated cataract and a good outcome (i.e. presenting vision 6/18 or better) as a proportion of those having operable plus operated cataract. As for CSC, eCSC was calculated using the cut-off for operable cataract of worse than 6/60 pinhole visual acuity (eCSC_persons <6/60_)_._

eCSC=[(a+b)/(x+y+z)]*100(%)

where

*a* = individuals with unilateral pseudo/aphakia achieving presenting visual acuity of 6/18 or better in the operated eye and operable cataract in the other eye;
*b* = individuals with bilateral pseudo/aphakia achieving presenting visual acuity of 6/18 or better in at least one eye;
*x*, *y* and *z* as above for CSC.

### Data analysis

Data analysis was performed in Stata 12.0 (StataCorp LP, TX) and SAS 9.4 (SAS Institute Inc, Cary, NC). The codes used to derive the variables are provided in S1 Table.

CSC_,_ eCSC_,_ CSO_Good_ and CSO_Poor_ were calculated for each country. Observed CSO_Good_ and CSO_Poor_ were plotted against CSC for each country, and compared to WHO targets[31] for CSO_Good_ (>80%) and CSO_Poor_ (<5%). To test the hypothesis that CSC can be used as a proxy indicator for CSO, linear regression was used to assess the association between i) CSC and CSO_Good_ and ii) CSC and CSO_Poor,_ and correlation co-efficients were calculated. CSC and eCSC were then plotted together and the relative gap between them was calculated for each country (i.e. 1 –eCSC / CSC).

#### Inequality

Gender is the only social variable routinely collected in RAAB surveys. To examine inequality, CSC and eCSC were calculated separately for women and men. The absolute gender inequality (i.e. the difference between women and men) for each outcome was calculated for each country. A logistic regression model was developed to analyze the likelihood of i) worse CSC (i.e. unoperated compared to operated cataract); and ii) worse eCSC (i.e. unoperated or operated cataract with a borderline/poor outcome compared to operated and a good outcome) in women compared to men. The model adjusted for age and the clustering effect of country, and results are presented using odds ratios (OR) and 95% confidence intervals (CI).

#### Sensitivity analysis: Time since surgery

Participants who underwent surgery more recently may have better outcomes than those who had surgery longer ago. Possible reasons for this include that earlier surgery may have used less refined procedures, and post-operative complications and other causes of visual loss may have accrued over time. The chi squared test (χ^2^) was used to assess the statistical significance of the difference in the proportion of eCSC between surgeries undertaken in the three years preceding the survey and those undertaken more than 3 years prior to the survey. Any individual who had bilateral surgery across the two time periods was categorized based on the more recent surgery.

## Results

On April 1 2016 datasets were available from 21 countries for surveys undertaken between 2005 and 2013. A dataset from Burundi was excluded as only eight participants had undergone cataract surgery. In the remaining 20 surveys, the number of participants ranged from 1,787–4,868 (total 67,337; median 3,170 inter-quartile range [IQR] 2,980–3,800) and the number of cataract surgeries ranged from 78–530 (total 5,474 in 3,795 people; median 239 IQR 164–390; S2 Table).

**Fig 1:**
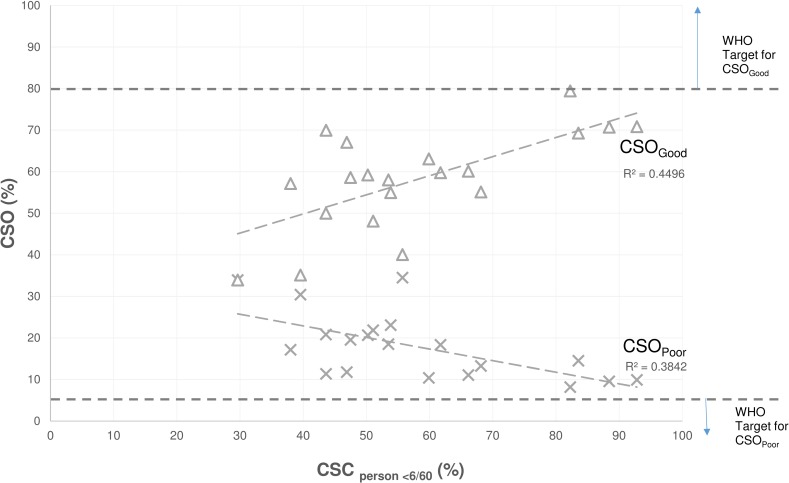
Proportion of operated eyes with presenting visual acuity of 6/18 or better (CSO_Good_) or worse than 6/60 (CSO_Poor_) plotted against observed cataract surgical coverage (CSC_persons <6/60_, %) in 20 countries, 2005–2013. WHO Targets established in 1998.[31].

The WHO targets[31] for CSO_Good_ (>80%) and CSO_Poor_ (<5%) were not met in any country, although the extent to which targets were not met varied substantially between countries (Fig 1). There was moderate association between CSC and both outcomes—every 1% increase in CSC was associated with a 0.46% increase in CSO_Good_ (p = 0.0012) and a 0.28% decrease in CSO_Poor_ (p = 0.0036). The R^2^ values indicate that CSC alone explained 45% of the variation in CSO_Good_ and 38% of the variation in CSO_Poor_.

**Fig 2:**
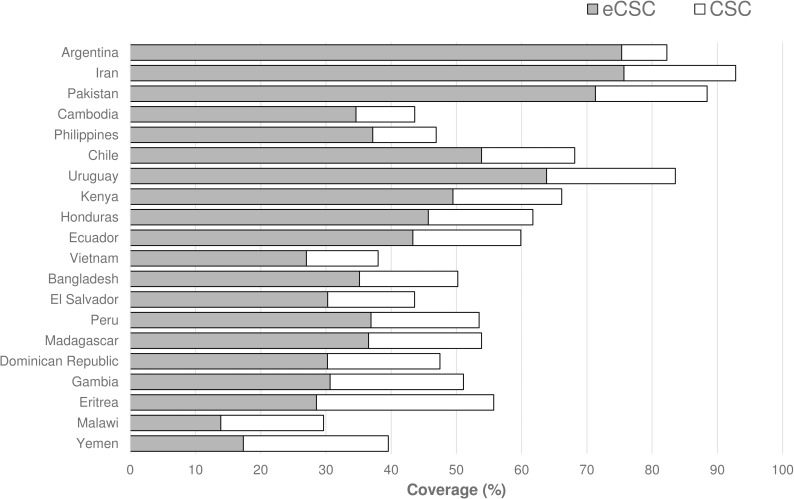
Cataract surgical coverage (CSC) and effective cataract surgical coverage (eCSC; persons <6/60, %) in 20 countries, 2005–2013. Arranged in ascending order of relative gap between CSC and eCSC (i.e. 1 –eCSC / CSC); the gap is smallest for Argentina and largest for Yemen.

The median eCSC (36.7% IQR 30.2–50.6%) was approximately one-third lower than the median CSC (53.7% IQR 46.1–66.6%) (Fig 2 and S2 Table). When arranged in order of the relative gap between CSC and eCSC, the surveys in Argentina, Iran and Pakistan revealed high levels of coverage (CSC 82.3%, 92.8% and 88.4% respectively) as well as relatively high levels of good outcomes (eCSC was 92%, 82% and 81% of the CSC value respectively). Next in the figure are Cambodia and the Philippines, two settings with only moderate coverage levels (43.6% and 46.9% respectively), but reasonably low gaps between CSC and eCSC, with eCSC being 79% of the CSC values in both locations. At the bottom of the figure, the countries with the largest relative gap between CSC and eCSC were Yemen (eCSC was 44% of the CSC value), Malawi (47%) and Eritrea (51%).

**Fig 3:**
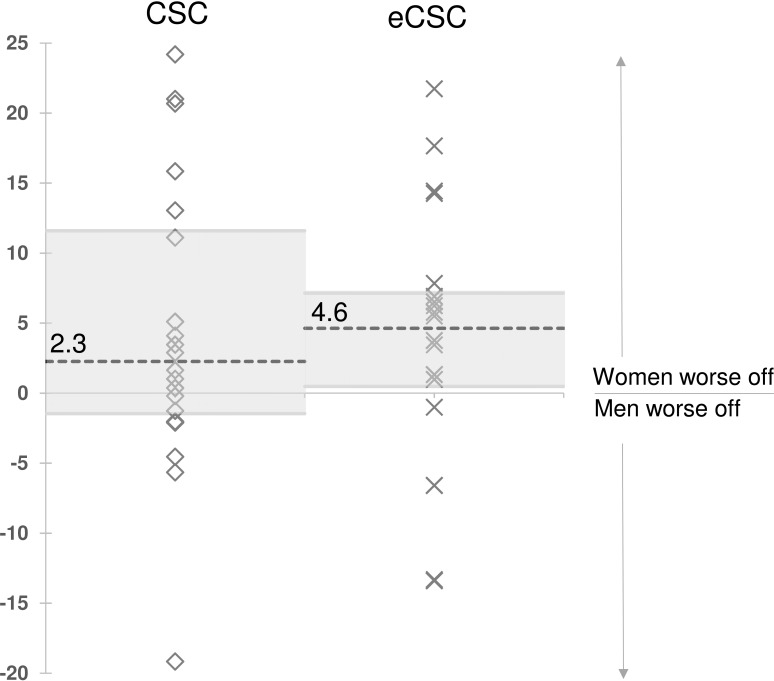
Absolute inequality between women and men in cataract surgical coverage (CSC_persons <6/60_), and effective cataract surgical coverage (eCSC_persons <6/60_) in 20 countries, 2005–2013. Absolute inequality is the difference between women and men (e.g. CSC in men–CSC in women); a positive value indicates women are worse off. Horizontal dashed lines and labels indicate the median values of all studies. Grey shading indicates the inter-quartile range (middle 50% of studies).

Gender inequality was present for both CSC and eCSC. Despite large variation between countries, on average women were worse off than men for both indicators (Fig 3). Gender inequality remained after controlling for possible age differences between women and men—logistic regression showed women were more likely than men to have worse CSC (OR 1.3, 95%CI 1.1–1.6) and worse eCSC (OR 1.3, 95%CI 1.0–1.5). The average level of inequality in eCSC (median 4.6% IQR 0.5–7.1%) was slightly higher than CSC (median 2.3% IQR -1.5–11.6%).

### Sensitivity analysis

Overall 2,086 of the 3,795 people who underwent surgery (55.0%) had done so in at least one eye within three years of the survey. There was no difference in the proportion of eCSC (*a*+*b*) between surgeries undertaken ≤3 years versus >3 years before the survey (43.1% versus 42.4% respectively; χ^2^ = 0.148, p = 0.700).

## Discussion

Despite moderate correlation between coverage (CSC) and quality (CSO) of cataract services (Fig 1), the results presented here confirm that in the context of UHC, it is insufficient to monitor coverage without also monitoring quality. Ideally all individuals with bilateral severe visual impairment from cataract would undergo cataract surgery and have their sight restored; i.e. 100% coverage with 100% success. All studies included in this analysis reveal gaps from this ideal. In most locations, cataract surgery failed to achieve the desired visual outcome, regardless of the coverage level. It appears there are some settings—shown in the bottom half of Fig 2—where people face the ‘double-disadvantage’ of low levels of service coverage, and low likelihood of a good visual outcome. However, this double-disadvantage is not universal, with surveys in Cambodia and the Philippines revealing relatively high levels of good visual outcomes in the context of only moderate coverage levels (Fig 2). Concurrent assessment of CSC and eCSC in this way allows a more nuanced policy response compared to assessment of CSC in isolation.[8]

Gender inequality in CSC is well known,[32] and our results confirm the tendency for women to have lower coverage than men (Fig 3). We also found higher levels of inequality in eCSC compared to CSC, highlighting a compounding of disadvantage for women, who tend to fare worse than men in quality of visual outcomes[33] in addition to lower coverage.

Inequality in CSC has also been reported across socio-economic status (SES),[34] and likely exists for other social factors. Disaggregated data are required to monitor inequality, and the UEH Action plan calls for CSC to be disaggregated by age, gender and urban/rural domicile.[14] For UHC, the minimum recommendation is to monitor inequality across gender, SES, and urban/rural domicile,[35] and we propose that eCSC is also monitored by these three factors as a minimum.

eCSC is a population-based indicator that can be used alongside CSC to promote quality improvement of cataract surgery at the district and national level. Tools already exist to assess cataract surgical outcome in clinical settings,[36, 37] and it has been shown that monitoring activities improve outcomes.[38] eCSC provides different information to these clinical tools, and should be used in conjunction with them to monitor services. For example, clinical tools can collect pre, peri- and post-operative findings to provide more accurate reasons for poor outcomes compared to assessments made during RAAB surveys. CSC and eCSC complement clinical tools by summarising the real-world results in a given population to understand access and quality of cataract surgery experienced by individuals and communities.

Adoption of eCSC at the global and national level may also lead to more focus on the quality of services, and exploration of factors contributing to low eCSC results could highlight priorities for intervention. For example, in most parts of the world aphakia has been superseded by pseudophakia due to better visual outcomes,[17] and tends to be prevalent among those undergoing surgery longer ago. However, aphakia remains an important contributor to poor outcomes in some settings. For example, eCSC in Yemen was very low (17.3%) and study reports available on the RAAB Repository (summarized in S3 Table) show that 42% of all surgeries in Yemen resulted in aphakia, compared to <1% of surgeries in Argentina, 3% in Kenya and 9% in Malawi.[30] High rates of aphakia persist in Yemen, with 31% of surgeries undertaken within three years of the 2009 survey resulting in aphakia. Understanding why this technique persists in some settings, and identifying strategies to increase the proportion of surgeries where IOLs are used will likely improve post-operative visual acuity and thus eCSC.

In addition to being a valuable UEH indicator, eCSC meets many of the criteria of UHC tracer indicators[26] as blindness and visual impairment from cataract are prevalent in people over the age of 50 years,[12] and have clear diagnostic criteria and effective treatment.[17] Also, data collection can readily occur through rapid, low-cost population-based surveys[29] and the UEH Action Plan has called for Member States to undertake more RAAB surveys to plan and monitor eye care services.[14] Through the RAAB Repository an increasing amount of comparable data are available from different settings,[30] including examples of national follow-up surveys that monitor change over time.[39] eCSC can be generated retrospectively from data previously collected, as demonstrated here. In future, it can be calculated easily from standard data collected in RAAB and other visual impairment surveys, and to ensure consistent and comparable results, the calculation of eCSC could be incorporated into the automated RAAB analysis.

One of the current shortcomings for eCSC to be a UHC tracer indicator is the lack of national-level data from many countries. This may soon be overcome through the ongoing development of a smartphone application (http://www.peekvision.org/) to undertake comprehensive eye examinations. As demonstrated here, the calculation of eCSC requires visual acuity assessment and detection of operable and operated cataract. There has already been a call for visual acuity assessment to be added to UHC monitoring tools.[7] “Peek” has already been validated for visual acuity assessment,[40] and found to be easy to use by general health workers.[41] Identifying operated and operable cataract currently requires an eye health worker with relatively extensive skills and experience, but Peek will enable general health workers to undertake this assessment using the smartphone and calling on remote support when necessary. This advance will allow eCSC data collection to occur within general household surveys, and vastly increase the availability of national-level eCSC data.

It is recommended that each country set its own UHC targets based on local priorities and realities,[42] and the same is true for eCSC. At the global level, targets can be established by combining coverage and outcome targets, as shown in Table 1. Of the 20 surveys analysed, four (20%) met these targets, identifying ‘good’ (Iran 75.7%, Argentina 75.3%, Pakistan 71.3%) or ‘satisfactory’ (Uruguay 63.8%) eCSC results. If eCSC is monitored in conjunction with CSC (as depicted in Fig 2) it will be possible to identify whether coverage, outcome, or both need improvement. Our sensitivity analysis showed that the time since surgery occurred (≤3 versus >3 years) did not alter eCSC results, so all surgeries can be included in the calculation of eCSC.

**Table 1:** Proposed global targets for effective cataract surgical coverage (eCSC)

*Coverage (CSC) Target (%)*		*Outcome (CSO*_*Good*_*) Target (%)*			*eCSC Target*	
95	X	95	=	90	≥90	Excellent
90	X	90	=	81	80–89	Very good
85	X	85	=	72	70–79	Good
80	X	80	=	64	60–69	Satisfactory

The components of eCSC provide a flexible indicator that can adapt to different health system contexts. Just as eCSC can be calculated using different visual acuity cut-offs for operable cataract, it can also be calculated using different visual acuity cut-offs for surgical outcome—e.g. in some settings 6/12 or better may be more appropriate than the more lenient 6/18 cut-off used here. Similarly, coverage with ‘good’ or ‘borderline’ visual outcomes can be calculated (i.e. 6/60 or better).

This analysis must be interpreted in the context of its limitations. Datasets were available from only 20 of the 74 countries in which RAAB surveys have been undertaken, the available datasets were not always the most recently undertaken study within a country, and less than half were based on a national sample frame. The findings therefore do not necessarily reflect the current national eCSC levels in these countries. What we have provided—by defining and demonstrating eCSC analysis—is a method of monitoring both coverage and quality of cataract services in one indicator, and a means of ongoing monitoring as more data become available. A further limitation was the inability to assess forms of inequality beyond gender, as other social variables are not routinely collected in RAAB surveys. More social variables will be included in RAABs in future, and more comprehensive assessment of inequality in eCSC (and other outcomes) will be possible.

We have defined and demonstrated eCSC, a measure that combines coverage with quality of cataract surgery, providing a valuable indicator for monitoring UEH. eCSC builds on the strengths of CSC―it can be calculated from data collected in existing eye health surveys, and in future will be incorporated into standard RAAB analysis outputs. It also has the potential to demonstrate inequities in service access and outcomes, and to be incorporated into general household surveys for more widespread data collection. As cataract development is an inevitable part of aging, eCSC provides an objective, easy-to-measure UHC indicator of services for the elderly.

## Supporting information

S1 Table Code used to derive variables for analysis. (PDF)

S2 Table Summary of included studies and results. (DOCX)

S3 Table Proportion of surgery resulting in aphakia in each location. (DOCX)

## References

[1] Donabedian A. The quality of care: how can it be assessed? JAMA. 1988;260(12):1743–8. 3045356 10.1001/jama.260.12.1743

[2] World Health Organization. What is universal coverage? 2016. Available from: http://www.who.int/health_financing/universal_coverage_definition/en/.

[3] Donabedian A. The seven pillars of quality. Arch Pathol Lab Med. 1990;114(11):1115–8. 2241519

[4] World Health Organization. Background paper for the technical consultation on effective coverage of health systems. Geneva: World Health Organization; 2001.

[5] Hogan D, Hosseinpoor AR, Boerma T. Developing an index for the coverage of essential health services Technical note for World health statistics 2016. Geneva: World Health Organization; 2016.

[6] Bryce J, Arnold F, Blanc A, Hancioglu A, Newby H, Requejo J, et al Measuring coverage in MNCH: new findings, new strategies, and recommendations for action. PLoS Med. 2013;10(5):e1001423 10.1371/journal.pmed.1001423 23667340 PMC3646206

[7] Boerma T, AbouZahr C, Evans D, Evans T. Monitoring intervention coverage in the context of universal health coverage. PloS Med. 2014;11(9):e1001728 10.1371/journal.pmed.1001728 25243586 PMC4171108

[8] Ng M, Fullman N, Dieleman JL, Flaxman AD, Murray CJ, Lim SS. Effective coverage: a metric for monitoring universal health coverage. PloS Med. 2014;11(9):e1001730 10.1371/journal.pmed.1001730 25243780 PMC4171091

[9] Hodgins S, D'Agostino A. The quality–coverage gap in antenatal care: toward better measurement of effective coverage. Glob Health Sci Pract. 2014;2(2):173–81. 10.9745/GHSP-D-13-00176 25276575 PMC4168625

[10] Campbell J, Buchan J, Cometto G, David B, Dussault G, Fogstad H, et al Human resources for health and universal health coverage: fostering equity and effective coverage. Bull World Health Organ. 2013;91(11):853–63. 10.2471/BLT.13.118729 24347710 PMC3853950

[11] World Health Organization and International Bank for Reconstruction and Development/The World Bank. Monitoring progress towards universal health coverage at country and global levels: A Framework. Discussion Paper. Geneva: World Health Organization, 2013.

[12] Bourne RR, Stevens GA, White RA, Smith JL, Flaxman SR, Price H, et al Causes of vision loss worldwide, 1990–2010: a systematic analysis. Lancet Glob Health. 2013;1(6):e339–49. 10.1016/S2214-109X(13)70113-X 25104599

[13] He W, Goodkind D, Kowal P. An Aging World: 2015. Washington, DC: U.S. Census Bureau; 2016.

[14] World Health Organization. Universal Eye Health: A global action plan 2014–2019. Geneva: World Health Organization, 2013.

[15] World Health Assembly. Towards universal eye health: A global action plan 2014–2019. Resolution 66.4, 24 May 2013. Geneva: World Health Assembly, 2013.

[16] Grimes CE, Henry JA, Maraka J, Mkandawire NC, Cotton M. Cost-effectiveness of surgery in low-and middle-income countries: a systematic review. World J Surg. 2014;38(1):252–63. 10.1007/s00268-013-2243-y 24101020

[17] Riaz Y, Mehta JS, Wormald R, Evans JR, Foster A, Ravilla T, et al Surgical interventions for age-related cataract. Cochrane Database Syst Rev. 2006;Issue 4. Art. No.: CD001323. 10.1002/14651858.CD001323.pub2 PMC7096771 17054134

[18] Essue BM, Li Q, Hackett ML, Keay L, Iezzi B, Tran KD, et al A Multicenter Prospective Cohort Study of Quality of Life and Economic Outcomes after Cataract Surgery in Vietnam: The VISIONARY Study. Ophthalmology. 2014;121(11):2138–46. 10.1016/j.ophtha.2014.05.014 25012931

[19] Finger RP, Kupitz DG, Fenwick E, Balasubramaniam B, Ramani RV, Holz FG, et al The impact of successful cataract surgery on quality of life, household income and social status in South India. PloS One. 2012;7(8):e44268 10.1371/journal.pone.0044268 22952945 PMC3432104

[20] Danquah L, Kuper H, Eusebio C, Rashid MA, Bowen L, Foster A, et al The Long Term Impact of Cataract Surgery on Quality of Life, Activities and Poverty: Results from a Six Year Longitudinal Study in Bangladesh and the Philippines. PloS One. 2014;9(4):e94140 10.1371/journal.pone.0094140 24747192 PMC3991652

[21] Mock CN, Donkor P, Gawande A, Jamison DT, Kruk ME, Debas HT. Essential surgery: key messages from Disease Control Priorities. Lancet. 2015;385(9983):2209–19. 10.1016/S0140-6736(15)60091-5 25662414 PMC7004823

[22] Henry JA, Bem C, Grimes C, Borgstein E, Mkandawire N, Thomas WE, et al Essential surgery: the way forward. World J Surg. 2015;39(4):822–32. 10.1007/s00268-014-2937-9 25566979

[23] Meara JG, Leather AJ, Hagander L, Alkire BC, Alonso N, Ameh EA, et al Global Surgery 2030: evidence and solutions for achieving health, welfare, and economic development. Lancet. 2015;386(9993):569–624. 10.1016/S0140-6736(15)60160-X 25924834

[24] Limburg H, Foster A, Gilbert C, Johnson G, Kyndt M, Myatt M. Routine monitoring of visual outcome of cataract surgery. Part 2: Results from eight study centres. Br J Ophthalmol. 2005;89(1):50–2. 10.1136/bjo.2004.045369 15615746 PMC1772465

[25] Limburg H, Foster A. Cataract Surgical Coverage: an indicator to measure the impact of cataract intervention programmes. Community Eye Health. 1998;11(25):3–6. PMC1706035 17492015

[26] World Health Organization and The World Bank. Tracking universal health coverage: first global monitoring report. Geneva: World Health Organization, 2015.

[27] Dineen B, Foster A, Faal H. A proposed rapid methodology to assess the prevalence and causes of blindness and visual impairment. Ophthalmic Epidemiol. 2006;13(1):31–4. 10.1080/09286580500473787 16510344

[28] Mathenge W, Bastawrous A, Foster A, Kuper H. The Nakuru posterior segment eye disease study: methods and prevalence of blindness and visual impairment in Nakuru, Kenya. Ophthalmology. 2012;119(10):2033–9. 10.1016/j.ophtha.2012.04.019 22721919

[29] Kuper H, Polack S, Limburg H. Rapid assessment of avoidable blindness. Community Eye Health. 2006;19(60):68–9. 17515970 PMC1871676

[30] Health Information Services. RAAB repository 2014. Available from: http://www.raabdata.info/.

[31] World Health Organization. Informal Consultation on Analysis of Blindness Prevention Outcomes. Geneva: World Health Organization, 1998 Contract No.: WHO/PBL/98.68.

[32] Lewallen S, Mousa A, Bassett K, Courtright P. Cataract surgical coverage remains lower in women. Br J Ophthalmol. 2009;93(3):295–8. 10.1136/bjo.2008.140301 19091848

[33] Ahmad K, Zwi AB, Tarantola DJ, Soomro AQ, Baig R, Azam SI. Gendered Disparities in Quality of Cataract Surgery in a Marginalised Population in Pakistan: The Karachi Marine Fishing Communities Eye and General Health Survey. PloS One. 2015;10(7):e0131774 10.1371/journal.pone.0131774 26186605 PMC4506126

[34] Cockburn N, Steven D, Lecuona K, Joubert F, Rogers G, Cook C, et al Prevalence, Causes and Socio-Economic Determinants of Vision Loss in Cape Town, South Africa. PLoS One. 2012;7(2):e30718 10.1371/journal.pone.0030718 22363476 PMC3282720

[35] Hosseinpoor AR, Bergen N, Koller T, Prasad A, Schlotheuber A, Valentine N, et al Equity-oriented monitoring in the context of universal health coverage. PLoS Med. 2014;11(9):e1001727 10.1371/journal.pmed.1001727 25243463 PMC4171107

[36] Congdon N, Suburaman G-B, Ravilla T, Varga B, Resnikoff S, McLeod J, et al Transforming research results into useful tools for global health: BOOST. Lancet Glob Health. 2016;4(2):e96 10.1016/S2214-109X(15)00267-3 26823227

[37] Limburg H, Foster A, Gilbert C, Johnson G, Kyndt M. Routine monitoring of visual outcome of cataract surgery. Part 1: Development of an instrument. Br J Ophthalmol. 2005;89(1):45–9. 10.1136/bjo.2004.045351 15615745 PMC1772455

[38] Yorston D, Gichuhi S, Wood M, Foster A. Does prospective monitoring improve cataract surgery outcomes in Africa? Br J Ophthalmol. 2002;86(5):543–7. 11973251 10.1136/bjo.86.5.543 PMC1771115

[39] Duerksen R, Limburg H, Lansingh VC, Silva JC. Review of blindness and visual impairment in Paraguay: changes between 1999 and 2011. Ophthalmic Epidemiol. 2013;20(5):301–7. 10.3109/09286586.2013.821497 24070101

[40] Bastawrous A, Rono HK, Livingstone IA, Weiss HA, Jordan S, Kuper H, et al Development and validation of a smartphone-based visual acuity test (peek acuity) for clinical practice and community-based fieldwork. JAMA Ophthalmol. 2015;133(8):930–7. 10.1001/jamaophthalmol.2015.1468 26022921 PMC5321502

[41] Lodhia V, Karanja S, Lees S, Bastawrous A. Acceptability, Usability, and Views on Deployment of Peek, a Mobile Phone mHealth Intervention for Eye Care in Kenya: Qualitative Study. JMIR Mhealth Uhealth. 2016;4(2):e30 10.2196/mhealth.4746 27160779 PMC4877502

[42] World Health Organization. Making fair choices on the path to universal health coverage. Final report of the WHO Consultative Group on Equity and Universal Health Coverage. Geneva: World Health Organization, 2014.

