# Supplementary material for: Effective cataract surgical coverage: An indicator for measuring quality-of-care in the context of Universal Health Coverage
Source: PLoS One. 2017 Mar 1;12(3):e0172342. doi: 10.1371/journal.pone.0172342 (PMC5382971; doi:10.1371/journal.pone.0172342)
Supplement: S1 Table — (PDF) [file pone.0172342.s001.pdf]

S1 Table: Code used to derive variables for analysis

Variable	Definition	Code from RAAB dataset
x	individuals with unilateral pseudo/aphakia (i.e. operated cataract) and operable cataract in the other eye	(bvare >2 & causere=3 & lere=2 & lele ≥3 & lele ≤5) OR<br>(bvale >2 & causele=3 & lele=2 & lere ≥3 & lere ≤ 5)
y	individuals with bilateral pseudo/aphakia, regardless of visual acuity	lere≥3 & lere≤5 & lele≥3 & lele ≤5
z	individuals with bilateral operable cataract	bvare > 2 & lere = 2 & bvale>2 & lele=2
a	individuals with unilateral pseudo/aphakia achieving presenting visual acuity of 6/18 or better in the operated eye and operable cataract in the other eye	(bvale >2 & causele=3 & lele=2 & lere ≥3 & lere≤5 & pvare=1) OR<br>(bvare >2 & causere=3 & lere=2 & lele ≥3 & lele ≤5 & pvale=1)
b	individuals with bilateral pseudo/aphakia achieving presenting visual acuity of 6/18 or better in at least one eye	lere≥3 & lere≤5 & lele≥3 & lele ≤5 & (pvare=1 OR pvale=1)
CSO_{Good}	presenting visual acuity of 6/18 or better in the operated eye of a person who had undergone unilateral cataract surgery, and the better eye of a person who had undergone bilateral cataract surgery	(lere≥3 & lere≤5 & lele≥3 & lele ≤5 & [pvare=1 OR pvale=1]) OR<br>(lere≥3 & lere≤5 & [lele<3 OR lele >5] & pvare=1) OR<br>(lele≥3 & lele≤5 & [lere<3 OR lere >5] & pvale=1)
CSO_{Poor}	presenting visual acuity of worse than 6/60 or better in the operated eye of a person who had undergone unilateral cataract surgery, and the better eye of a person who had undergone bilateral cataract surgery	(lere≥3 & lere≤5 & lele≥3 & lele ≤5 & pvare>2 & pvale>2) OR<br>(lere≥3 & lere≤5 & [lele<3 OR lele >5) & pvare>2) OR<br>(lele≥3 & lele≤5 & [lere<3 OR lere >5] & pvale>2)

Variables used to calculate:
Cataract surgical coverage: $CSC_{person<6/60} = [(x + y)/(x + y + z)]*100$ (%)
Effective CSC: $eCSC_{person<6/60} = [(a + b)/(x + y + z)]*100$ (%)

bvare/ bvale: pinhole visual acuity right/ left eye

causere/ causele: primary cause of reduced visual acuity right/ left eye

lere/ lele: Lens examination right/ left eye

pvare/ pvale: presenting visual acuity right/ left eye
