# Supplementary material for: Effective cataract surgical coverage: An indicator for measuring quality-of-care in the context of Universal Health Coverage
Source: PLoS One. 2017 Mar 1;12(3):e0172342. doi: 10.1371/journal.pone.0172342 (PMC5382971; doi:10.1371/journal.pone.0172342)
Supplement: S2 Table — (DOCX) [file pone.0172342.s002.docx]

S2 Table: Summary of included studies and results

| **Country** | **Year of study** | **Participants**  **n** | **Cataract surgeries  n** | **CSO_Good_**  **%** | **CSO_Poor_**  **%** | **CSC _persons_ _<6/60_ %** | | | **eCSC _persons <6/60_ %** | | |
| --- | --- | --- | --- | --- | --- | --- | --- | --- | --- | --- | --- |
|  |  |  |  |  |  | **Women** | **Men** | **Total (95% CI)** | **Women** | **Men** | **Total (95% CI)** |
| Iran | 2009 | 2,819 | 530 | 70.8 | 9.9 | 92.3 | 93.3 | 92.8 (88.6-95.8) | 75.2 | 76.2 | 75.7 (69.5-81.2) |
| Argentina* | 2013 | 3,770 | 455 | 79.4 | 8.2 | 83.1 | 81.1 | 82.3 (76.7-87.0) | 75.7 | 74.7 | 75.3 (69.2-80.7) |
| Pakistan | 2013 | 3,084 | 456 | 70.7 | 9.6 | 88.2 | 88.6 | 88.4 (83.4-92.4) | 67.6 | 74.6 | 71.3 (64.8-77.2) |
| Uruguay* | 2011 | 3,729 | 351 | 69.3 | 14.5 | 82.2 | 86.3 | 83.6 (76.7-89.1) | 63.4 | 64.7 | 63.8 (55.6-71.4) |
| Chile | 2006 | 2,915 | 140 | 55.1 | 13.3 | 75.9 | 56.8 | 68.1 (57.5-77.5) | 59.3 | 45.9 | 53.8 (43.1-64.4) |
| Kenya | 2011 | 3,124 | 299 | 60.1 | 11.1 | 57.7 | 78.7 | 66.1 (58.8-72.9) | 42.3 | 60.0 | 49.5 (42.1-56.9) |
| Honduras* | 2013 | 2,999 | 240 | 59.8 | 18.3 | 60.6 | 63.5 | 61.7 (53.8-69.2) | 43.4 | 49.2 | 45.7 (37.8-53.7) |
| Ecuador* | 2008/9 | 4,012 | 444 | 63.1 | 10.4 | 60.5 | 59.3 | 59.9 (54.2-65.3) | 46.7 | 40.1 | 43.3 (37.8-49.0) |
| Philippines | 2006 | 3,177 | 121 | 67.1 | 11.8 | 46.5 | 48.1 | 46.9 (37.5-56.5) | 33.7 | 48.1 | 37.2 (28.3-46.8) |
| Peru* | 2011 | 4,849 | 238 | 58.0 | 18.5 | 55.7 | 50.0 | 53.5 (46.1-60.8) | 34.8 | 40.3 | 36.9 (30.0-44.2) |
| Madagascar | 2011 | 3,157 | 129 | 54.9 | 23.1 | 50.0 | 61.1 | 53.8 (43.8-63.7) | 41.2 | 27.8 | 36.5 (27.3-46.6) |
| Bangladesh | 2005 | 4,868 | 228 | 59.2 | 20.7 | 51.0 | 48.9 | 50.2 (43.7-56.7) | 33.8 | 37.2 | 35.1 (29.1-41.6) |
| Cambodia | 2011/12 | 4,471 | 372 | 70.0 | 11.4 | 42.7 | 46.2 | 43.6 (38.3-49.0) | 33.6 | 37.4 | 34.6 (29.6-39.9) |
| Gambia* | 2007 | 2,922 | 257 | 48.1 | 21.9 | 43.1 | 63.7 | 51.1 (44.5-57.6) | 22.2 | 44.0 | 30.6 (24.8-37.0) |
| El Salvador* | 2011 | 3,399 | 216 | 50.0 | 20.8 | 43.7 | 43.5 | 43.6 (36.5-50.9) | 27.2 | 33.7 | 30.3 (23.9-37.2) |
| Dominican Republic* | 2008 | 3,873 | 172 | 58.6 | 19.5 | 50.0 | 45.5 | 47.5 (39.0-56.1) | 33.9 | 27.3 | 30.2 (22.7-38.6) |
| Eritrea* | 2008 | 3,163 | 468 | 40.1 | 34.5 | 53.4 | 58.5 | 55.7 (50.5-60.9) | 27.9 | 29.3 | 28.5 (24.0-33.4) |
| Vietnam† | 2007 | 1,787 | 90 | 57.1 | 17.1 | 33.8 | 46.9 | 38.0 (28.5-48.3) | 25.0 | 31.3 | 27.0 (18.6-36.8) |
| Yemen | 2009 | 1,789 | 190 | 35.1 | 30.4 | 32.2 | 48.1 | 39.6 (33.1-46.3) | 10.7 | 25.0 | 17.3 (12.6-22.9) |
| Malawi | 2009/10 | 3,430 | 78 | 33.9 | 33.9 | 20.0 | 44.2 | 29.6 (21.2-39.2) | 10.8 | 18.6 | 13.9 (8.0-21.9) |
| Median |  | 3,170 | 239 | 58.9 | 17.7 | 52.2 | 57.6 | 53.7 | 34.3 | 40.2 | 36.7 |
| IQR |  | 2,980-3,800 | 164-390 | 53.7-67.6 | 11.3-21.1 | 43.5-64.4 | 47.8-67.5 | 46.1-66.6 | 27.8-49.8 | 30.8-51.9 | 30.2-50.6 |

CSO: Cataract surgical outcome; presenting vision in the operated eye; for those having bilateral surgery, it is vision in the better eye; CSO_Good_ is vision of 6/18 or better; CSO_Poor_ is vision worse than 6/60.

CSC _persons <6/60_: the number of people in a defined population with operated cataract as a proportion of those having operable plus operated cataract.

eCSC _persons <6/60_: the number of people in a defined population with operated cataract and a good outcome (i.e. presenting vision 6/18 or better) as a proportion of those having operable plus operated cataract.

IQR: Inter-quartile range

95% CI: 95% confidence interval

*sample drawn from national population

†Vietnam was the only country with more than one dataset available from the same year; the survey from Binh Phuoc was randomly selected for inclusion.
