# Supplementary material for: Effective cataract surgical coverage: An indicator for measuring quality-of-care in the context of Universal Health Coverage
Source: PLoS One. 2017 Mar 1;12(3):e0172342. doi: 10.1371/journal.pone.0172342 (PMC5382971; doi:10.1371/journal.pone.0172342)
Supplement: S3 Table — (DOCX) [file pone.0172342.s003.docx]

S3 Table: Proportion of surgery resulting in aphakia in each location

| **Country** | **Year of study** | **Participants**  **n** | **Cataract surgeries  n** | **Proportion of surgery resulting in aphakia (% eyes)** | |
| --- | --- | --- | --- | --- | --- |
|  |  |  |  | **All surgery** | **Surgery in the three years before the survey** |
| Yemen | 2009 | 1,789 | 190 | 41.6 | 30.5 |
| Bangladesh | 2005 | 4,868 | 228 | 45.2 | 19.8 |
| Philippines | 2006 | 3,177 | 121 | 16.5 | 12.3 |
| Eritrea* | 2008 | 3,163 | 468 | 25.0 | 11.8 |
| Gambia* | 2007 | 2,922 | 257 | 25.3 | 7.6 |
| Peru* | 2011 | 4,849 | 238 | 4.2 | 5.9 |
| Iran | 2009 | 2,819 | 530 | 7.2 | 4.5 |
| Malawi | 2009/10 | 3,430 | 78 | 9.0 | 4.2 |
| Dominican Republic* | 2008 | 3,873 | 172 | 5.8 | 2.8 |
| El Salvador* | 2011 | 3,399 | 216 | 4.6 | 2.0 |
| Vietnam† | 2007 | 1,787 | 90 | 16.7 | 1.8 |
| Cambodia | 2011/12 | 4,471 | 372 | 7.3 | 1.8 |
| Pakistan | 2013 | 3,084 | 456 | 6.4 | 1.7 |
| Madagascar | 2011 | 3,157 | 129 | 3.9 | 1.4 |
| Ecuador* | 2008/9 | 4,012 | 444 | 2.3 | 1.3 |
| Uruguay* | 2011 | 3,729 | 351 | 3.7 | 1.0 |
| Kenya | 2011 | 3,124 | 299 | 2.7 | 0.7 |
| Chile | 2006 | 2,915 | 140 | 3.6 | 0.0 |
| Honduras* | 2013 | 2,999 | 240 | 3.3 | 0.0 |
| Argentina* | 2013 | 3,770 | 455 | 0.7 | 0.0 |
| Median |  | 3,170 | 239 | 6.1 | 1.9 |
| IQR |  | 2,980-3,800 | 164-390 | 3.7-16.6 | 1.2-6.3 |

IQR: Inter-quartile range

*sample drawn from national population

†Vietnam was the only country with more than one dataset available from the same year; the survey from Binh Phuoc was randomly selected for inclusion.
